# Intracellular calcium response of primary cilia of tubular cells to modulated shear stress under oxidative stress

**DOI:** 10.1063/5.0010737

**Published:** 2020-07-02

**Authors:** Masatomo Chikamori, Hiroshi Kimura, Reiko Inagi, Jing Zhou, Masaomi Nangaku, Teruo Fujii

**Affiliations:** 1 Institute of Industrial Science, The University of Tokyo, Tokyo 153-8505, Japan; 2 Department of Mechanical Engineering, School of Engineering, Tokai University, Kanagawa 259-1292, Japan; 3 Department of Nephrology and Endocrinology, Graduate School of Medicine, The University of Tokyo, Tokyo 113-0033, Japan; 4 Center for Polycystic Kidney Disease Research and Renal Division, Department of Medicine, Brigham and Women's Hospital, Harvard Medical School, Boston, Massachusetts 02115, USA

## Abstract

Primary cilia of tubular cells are sensory organelles. Bending of the primary cilia with shear stress from urinary flow results in the elevation of intracellular calcium levels and activation of signaling pathways that maintain kidney function. Elongation of primary cilia is reported to occur due to oxidative stress, which is a major cause of ischemia-reperfusion injury and is accompanied by decreased kidney function. However, in the context of diminished kidney function, this elongation is yet to be investigated. In this study, we developed a new microfluidic device to monitor changes in the intracellular calcium levels while modulating shear stress on the cilia under different degrees of oxidative stress. The microfluidic device was designed to expose even shear stress in the observed area while supplying drugs in four different stepwise concentrations. The results showed that primary cilia were elongated by hydrogen peroxide, which induces oxidative stress. It was also observed that the elongated primary cilia were more sensitive to shear stress than those with normal morphology. This microfluidic device could, thus, be useful in the analysis of the morphology of the primary cilia, under low perfusion conditions.

## I. INTRODUCTION

Each vertebrate cell, excluding those of myeloid or lymphoid origin, has a primary cilium on the apical surface of the plasma membrane.^1^ Primary cilia sense mechanical or chemical stress.^2–4^ They contain signaling cascades, such as the non-canonical Wnt-PCP signaling pathway, for establishing apicobasal polarity.^5^ Primary cilia on tubular cells are bent by shear stress from urinary flow, and this results in calcium flow in the primary cilia through a complex of polycystin 1 and polycystin 2, transient receptor potential canonical 1 (TRPC1), or transient receptor potential vanilloid 4 (TRPV4). After calcium inflow, more calcium is released from the endoplasmic reticulum through polycystin 2 and ryanodine receptors or inositol 1,4,5-trisphosphate (IP3) receptors. Thus, intracellular calcium levels are increased, and cell proliferation is regulated through the Ras/Raf-1/MEK/ERK pathway.^6^

Autosomal dominant polycystic kidney disease (ADPKD) is the most common monogenic cause of renal failure.^7^ ADPKD patients have a mutation in PKD1 or PKD2, which encodes polycystin 1 and polycystin 2, respectively. In these patients, cilia bending does not modulate calcium levels resulting in abnormal proliferation and loss of cell polarity.^8,9^ Excessive cells and polarity depletion alter the alignment of tubules and cause the formation of cysts, which constitute a layer of tubular cells that enclose water within. Enlargement of cysts with time leads to strain on normal kidney tissues, causing gradual deterioration of kidney function.

Recent advances in microfluidic technologies have enabled the use of perfusion system chemicals to alter the length and function of primary cilia on tubular cells and study the effects. For instance, both cyclic AMP (cAMP) analog and protein kinase C (PKC) activator can elongate primary cilia. The increase in intracellular calcium levels induced by bending primary cilia by shear stress was more pronounced in cells exposed to the cAMP analog than in cells that were not exposed.^10^ On the other hand, exposing the PKC activator did not affect changes in the intracellular calcium in response to bending primary cilia.

Although these studies have demonstrated an increase in the intracellular calcium levels, the focus was mainly on providing constant shear stress during stimulation, in most cases, as strong as what is observed in perfusion culture. The elongation of primary cilia length is also observed under oxidative stress.^11,12^ Oxidative stress is a primary factor responsible for injury and impaired kidney function in ischemia-reperfusion injury.^13^ After an injury, single nephron glomerular filtration rates are often decreased,^14^ and shear stress inside the tubules are lowered. Therefore, it is desirable to develop a culture system that allows for regulation of the flow rate.

In this study, we examined the morphological changes in primary cilia induced by oxidative stress. Further, we modulated the strength of shear stress against primary cilia and observed the intracellular calcium levels.

## II. MATERIALS AND METHODS

### A. Design and fabrication

**FIG. 1.**
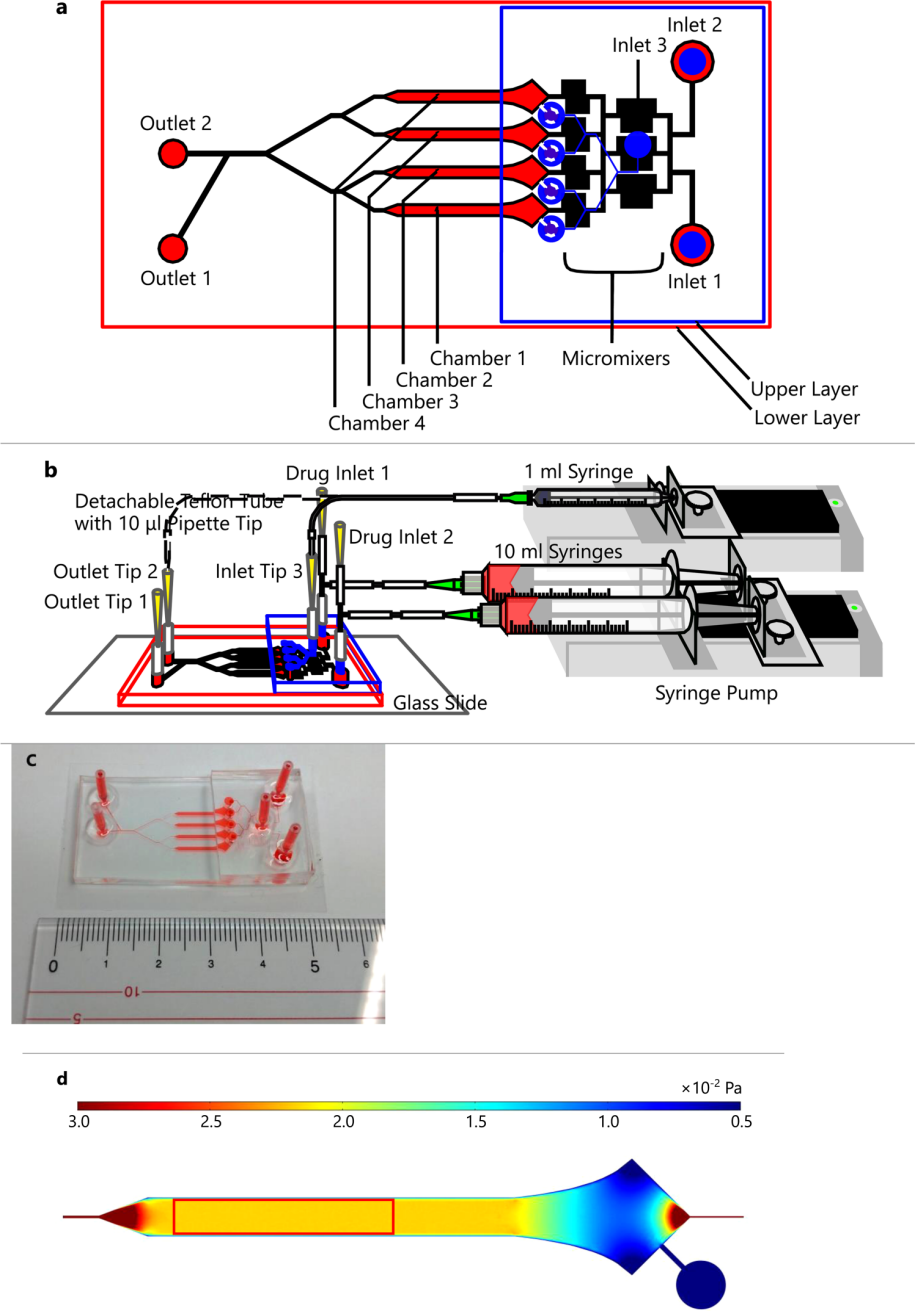
(a) Schematic of the designed microfluidic device (not to scale). (b) Assembly of the microfluidic device. The Teflon tube connected to a 1-ml syringe can be joined to outlet tip 2 or inlet tip 3. (c) The microfluidic device image. (d) The strength of shear stress on the surface of tubular cells. The red rectangle shows the observation area for primary cilia and intracellular calcium levels.

The microfluidic device was constructed by two polydimethylsiloxane (PDMS) layers and was assembled from 200 *μ*l pipet tips (BMT-200RS, BMBio, Japan), of which we only used the cones, a 10-*μ*l pipet tip (FG-103RS, Nippon Genetics Co., Ltd., Japan), Teflon tubes (UF-100-1 × 1.5-10M, MiSUMi, Japan), silicone tubes (9-869-02, AS ONE, Japan), mini T-shaped connectors (VFT106, ISIS Co., Ltd., Japan), and syringes (SS-01T or SS-10LZ, Terumo, Japan), as illustrated in Figs. 1(a)–1(c). The upper layer was used as inlets for culture medium (inlets 1 and 2), drug (drug inlets 1 and 2 to inlets 1 and 2), or immunofluorescent dye (inlet tip 3 to inlet 3). The lower layer was used for cell culture in four chambers (chambers 1–4) and for mixing drug solutions in micromixers to make stepwise concentration distributions. Perfusion culture was achieved by two 10-ml syringes connected to a motorized syringe pump (MFS-SP1, Microfluidic System Works Inc., Japan). Both 10-ml syringes connected to the syringe pump had the same flow rate. Aspiration for cell seeding and intracellular calcium levels observation were performed with a 1-ml syringe, which could be joined to inlet tip 3 or outlet tip 2, and was connected with another syringe pump.

The length of channels in micromixers was long enough for proteins with the molecular weight under 3300 g mol^−1^, which is over twofold of the molecular weights of drugs affecting tubular cells like antibiotics or diuretic drugs, to diffuse at 37 °C. The diffusion efficiency and length of channels were calculated using the following equations:^15,16^

$$\begin{array}{c} D≅\frac{8.34\times 10^{-6}\times T}{MW^{\frac{1}{3}}\times \mu } \end{array},$$ (1)

$$\begin{array}{c} L≅\frac{Q\times w}{D\times h}, \end{array}$$ (2)

where *D* is the diffusion efficiency (mm^2^ s^−1^), *T* is the temperature (K), MW is the molecular weight of solution (g mol^−1^), *μ* is the medium viscosity (mPa s), *L* is the length required for diffusion of the solution evenly, *Q* is the flow rate, *w* is the chamber width, and *h* is the height of the medium.

The chamber width and observation areas were configured to have uniform shear stress at any point in the observation area. Figure 1(d) shows the strength of shear stress on the apical surface of tubular cells. The observation area was restricted within the area marked by the red rectangle.

The film mask (System Advance, Japan), designed using AutoCAD 2018 (Autodesk, USA), was used to fabricate a 4-in. silicon wafer mold, patterned with SU-8 2100 (MicroChem Corp., USA) using photolithography. The height was set at 120 *μ*m. A mixture of polydimethylsiloxane (PDMS; SILPOT 184 W/C, Dow Corning Toray, Japan) and curing agent [10:1 (w/w)] was poured on the mold to 3-mm thickness and degassed for 15 min. The PDMS was cured at 75 °C for 150 min. After the removal of PDMS and punching of inlets and outlets, a glass slide (C035551, Matsunami Glass Ind., Ltd., Japan) and a lower layer of PDMS, followed by an upper layer and the lower layer of PDMS, were permanently bonded by oxygen plasma. Silicone tubes (9-869-02, AS ONE, Japan) were bonded to each inlet and outlet using PDMS.

### B. Cell culture, hydrogen peroxide exposure, and seeding

Renal cortical tubule epithelial cells of distal tubule origin, as evidenced by *Dolichos biflorus* lectin staining (RCTEC-DBA) cells,^17^ were grown at 37 °C at 5% CO_2_ and maintained in Dulbecco's modified Eagle's medium (10569-010, GIBCO, USA) containing 10% fetal bovine serum (FBS; 172012-500ML, Nichirei bioscience Inc., Japan), 50 IU/ml penicillin, and 50 *μ*g/ml streptomycin (15140-122, GIBCO, USA). RCTEC-DBA cells were a kind gift from Harvard University.

We used a microfluidic device for each experiment and observed the microfluidic device on time-lapse microscopy in an incubator (CCM-1.3XYZ/CO2, ASTEC, Japan) at 37 °C at 5% CO_2_. We injected 5 *μ*l of the cell suspension (6.0 × 10^4^ cells) into the outlet tip 2, aspirating from the inlet tip 3 at 1 *μ*l min^−1^ with a 1-ml syringe. After 15 min, we started to perfuse each chamber with DMEM containing 10% FBS at 1 *μ*l min^−1^ (which was equivalent to 0.025 Pa and is a quarter of the shear stress *in vivo*^18,19^), each chamber using two 10-ml syringes. During this waiting time of 15 min, we switched the Teflon tube connecting the 1-ml syringe from the inlet tip 3 to outlet tip 1. We injected 400 *μ*l of DMEM with and without 400 *μ*M hydrogen peroxide (H_2_O_2_; 081-04215, Wako, Japan) through drug inlets, withdrew the Teflon tubes using 10-ml syringes and exposed 0, 100, 300, and 400 *μ*M of H_2_O_2_ into chambers 1, 2, 3, and 4, respectively, 24 h after cell seeding. After injecting the H_2_O_2_ solution, we again perfused each chamber with DMEM containing 10% FBS at 1 *μ*l min^−1^ to each chamber for 48 h.

### C. Primary cilia observation

RCTEC-DBA cells were processed for immunofluorescence staining 48 h after H_2_O_2_ exposure with two primary antibodies for primary cilia [ARL 13B antibody (1711-1-AP, Proteintech, USA) and anti-acetylated α tubulin antibody (T7451-100ML, Sigma-Aldrich, USA)] and secondary antibodies [Alexa Fluor 488 (1711-1-AP, Proteintech) and Alexa Fluor 555 (ab150082, Abcam, UK)], and nuclei were counterstained with DAPI (KS042, Dojindo, Japan). The immunofluorescence procedure was performed by inducing the following solutions into inlet tip 3 sequentially by aspirating from outlet tip 2 at 2 *μ*l min^−1^ using a 1-ml syringe: 20 *μ*l of 4% paraformaldehyde (163-20145, Wako, Japan) for 10 min, 40 *μ*l of phosphate-buffered saline (PBS; 11482-15, Nacalai Tesque, Inc., Japan) for 20 min, 30 *μ*l of 0.3% Triton X (T8787, Sigma-Aldrich) for 15 min, 100 *μ*l of 3% bovine serum albumin (BSA; A9418-100G, Sigma-Aldrich) for 50 min, 120 *μ*l of PBS containing ARL-13B antibody and anti-acetylated α tubulin antibody for 60 min, 120 *μ*l of PBS containing Alexa Fluor 488, Alexa Fluor 555, and DAPI for 60 min, and 240 *μ*l of PBS for 120 min. Images of primary cilia were recorded as three-dimensional data using structured illumination microscopy (BZ-X710, Keyence Corporation, Japan), and length of primary cilia was measured after projecting two dimensions using ImageJ/FIJI (1.52h).^20^

### D. Intracellular calcium level observation

We injected DMEM containing 1% FBS, 0.5% Fluo 4-AM, 1.25 mM probenecid and 0.01% pluronic F-127 (Calcium Kit II - Fluo 4; CS32, Dojindo) to inlet tip 3 and exposed the Fluo 4-AM solution to RCTEC-DBA by aspirating from outlet tip 2 with a 1-ml syringe for 30 min. During the observation of the intracellular calcium level, we perfused DMEM containing 1% FBS and 1.25 mM probenecid by aspirating from outlet tip 2 with a 1-ml syringe incrementing the flow rate from 0.25 *μ*l min^−1^ to 0.65 *μ*l min^−1^ (equivalent to 0.00625 Pa and 0.01625 Pa, respectively) by 0.075 *μ*l min^−1^ or 0.125 *μ*l min^−1^ in each chamber. The interval time of exposure to the 0.25 *μ*l min^−1^ flow and other rates was 2 and 1 min, respectively. Using a 1-ml syringe, we could control and maintain the medium velocity (Fig. S1 in the supplementary material). We obtained images of the intensity of Fluo 4-AM every 12 s while increasing the shear stress gradually by modulating the flow rate of the DMEM containing 1% FBS and 1.25 mM probenecid.

Projections of the average intensity of stack of the first 2 min were acquired from the time-lapse images. We subtracted the average intensity image from each image, and manually set the threshold intensity to detect the brightened cells. Then, the threshold intensity areas were divided into cells using the watershed method and defined as regions of interest. We measured the time course of the intensity of each cell. The time-point of the increase in the intracellular calcium level was defined as the first time when the fluorescent intensity was over the regression line plus twofolds of the standard error acquired from the data during the first 2 min.

### E. Computational fluid dynamics simulation

We used a general-purpose simulation software (COMSOL Multiphysics 5.2.0.220, COMSOL Inc., Burlington, USA) to estimate the relationship between shear stress against the apical surface of tubular cells (i.e., the roots of primary cilia) and flow rate of perfused DMEM. To calculate the shear stress, we measured the thickness of the culture medium in chambers, which was obtained by subtracting the cell heights from chamber heights measured in advance by BZ-X710. All fluid materials were simulated as water at 37 °C.

### F. Statistical analysis

We used EZR 1.40 (a graphical user interface package for R statistical software^21^) for statistical analysis.^22^ Comparisons between four chambers were made using one-way ANOVA and post hoc test with the Bonferroni method. *P* < 0.05 was considered significant.

## III. RESULTS AND DISCUSSION

### A. Concentration gradient

**FIG. 2.**
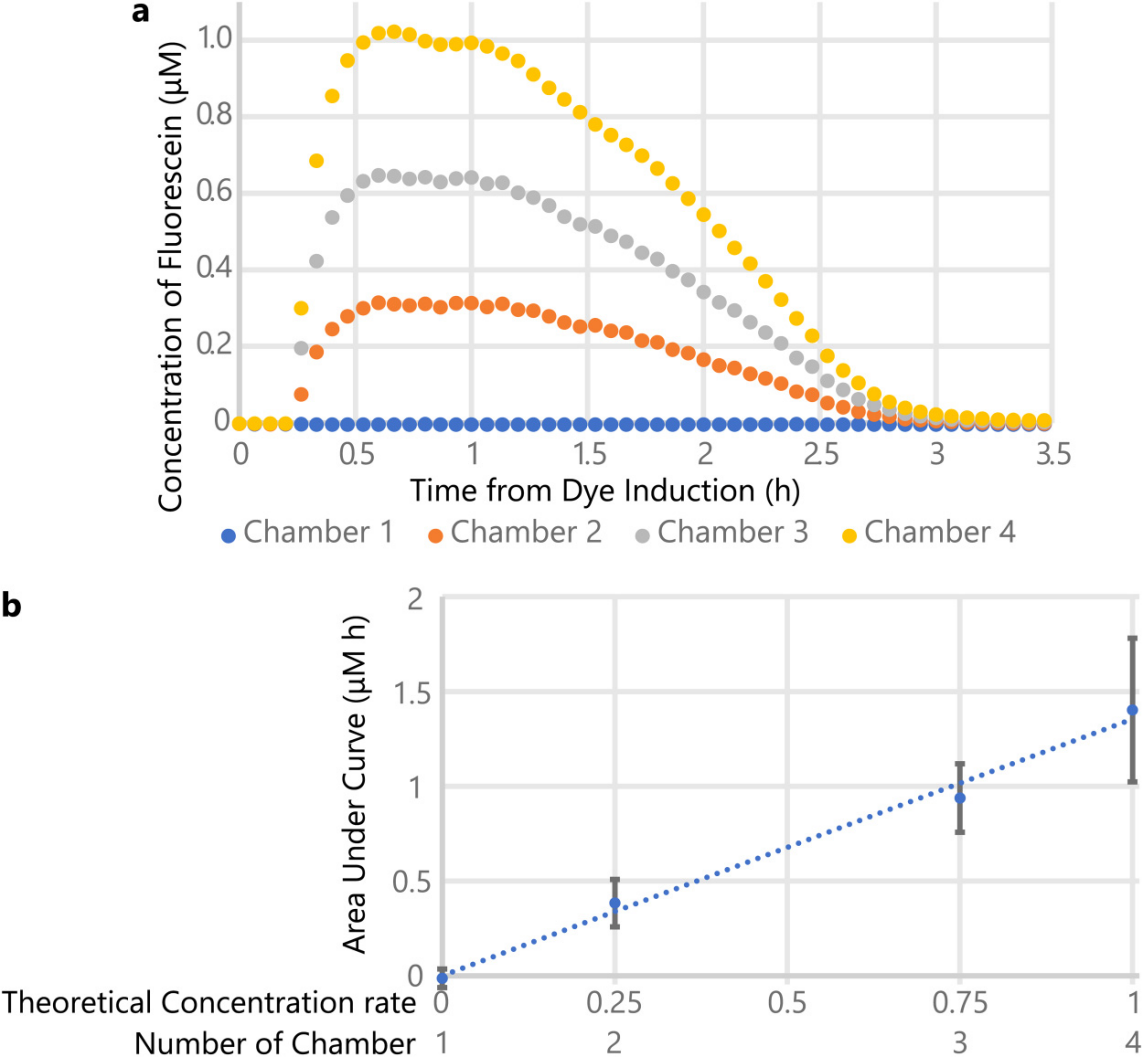
(a) The time course of the fluorescein concentration in each chamber since the medium containing fluorescein was induced (n = 1). The concentration of fluorescein was calculated by the concentration and intensity titration experiment. (b) The relationship between area under the curve in (a) and the theoretical concentration rate in each chamber (n = 6). Error bars represent standard deviation.

A culture medium containing 1.24 *μ*M fluorescein was used to confirm the function of micromixers. Figure 2(a) shows the fluorescein concentration in each chamber when 200 *μ*l of the medium with and without fluorescein was introduced from drug inlets 1 and 2, respectively, and each chamber was perfused at 1 *μ*l min^−1^. The fluorescein concentration was calculated from the regression line between immunofluorescent intensity and adjusted concentration, which were measured in advance using the microfluidic device (data not shown). Figure 2(b) shows the areas under the curve for fluorescein concentration in each chamber and the regression line. The measured areas under curve had a strong correlation (Pearson coefficient: 0.92, *p* < 0.01) with theoretical concentration distribution. These data validated the accuracy of drug mixing.

### B. Primary cilia elongation

**FIG. 3.**
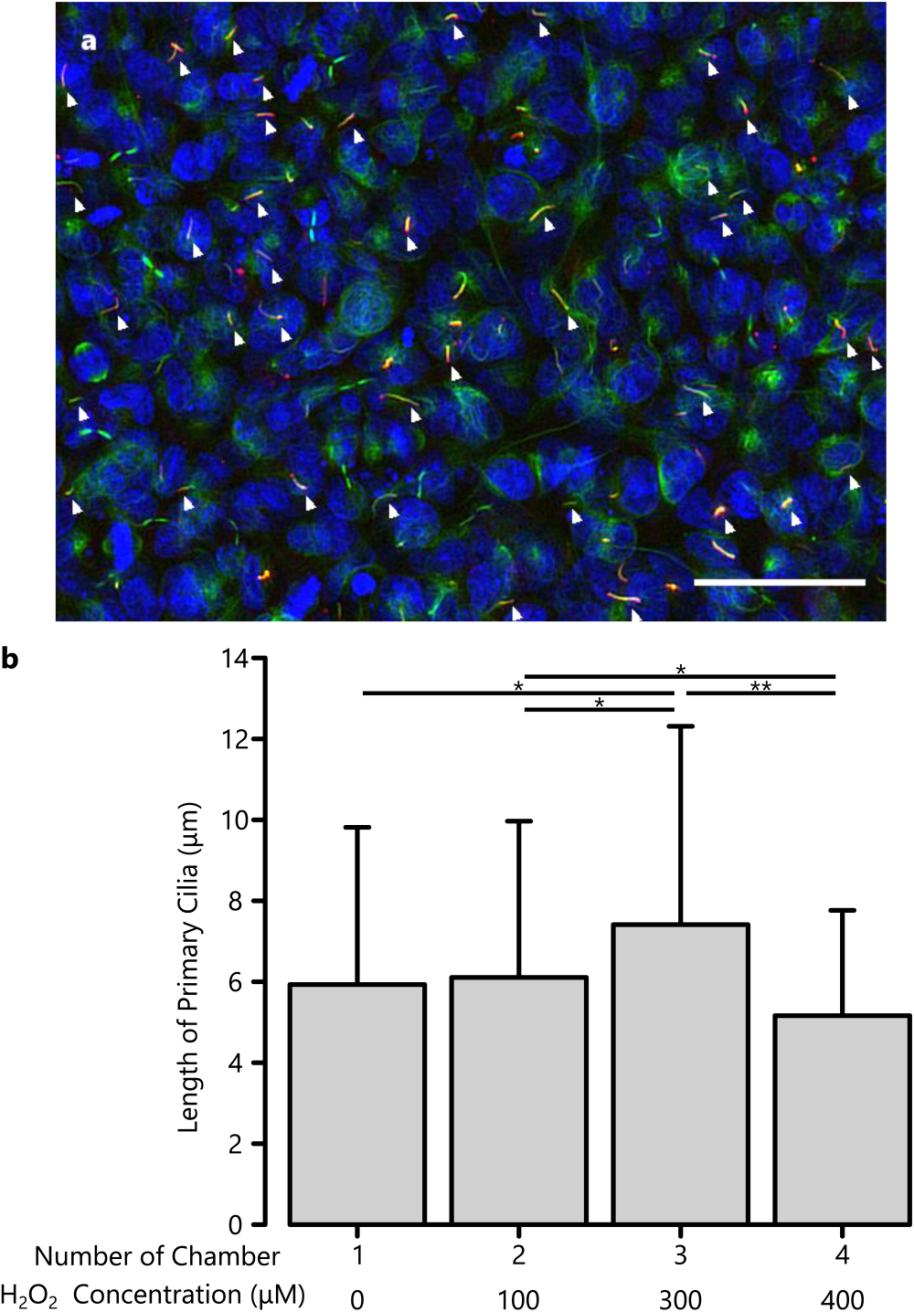
(a) The primary cilia (white arrows) stained with ARL 13B antibody (red) and anti-acetylated α tubulin antibody (green), and nuclei counterstained with DAPI. The scale bar is 50 *μ*m (b) The relationship between exposed H_2_O_2_ concentration and length of primary cilia. Error bars represent standard deviation. *, *p* < 0.05. **, *p* < 0.01.

Figure 3(a) shows the two-dimensionally projected image of immunofluorescent stained primary cilia. Observations were made in at least three of each chamber for the measurement of the length of primary cilia. Primary cilia were elongated approximately 1.25-fold after exposure to 300 *μ*M H_2_O_2_ [Fig. 3(b)], similar to previous findings in Petri dishes that reported this mechanism of primary cilia elongation related to extracellular signal-regulated kinase (ERK) activation.^11,12^ However, the cilia elongation effect did not show dose dependency with H_2_O_2_ concentration. This could be due to shear stress, which is reported to activate the nuclear factor E2-related factor 2 (Nrf2) pathway and regulate cytoprotective responses to oxidative stresses.^23^ Moreover, large variations in cilia length were possible between devices because cell densities between devices (not chambers) were not equal; thus, cell cycles were different between devices. Further, there were fewer primary cilia in chamber 4 than in other chambers. RCTEC-DBA cells were occasionally detached when the concentration of H_2_O_2_ was 400 *μ*M and the number of cells was decreased by approximately 25% compared to the numbers of cells in other chambers, indicating that at this concentration, H_2_O_2_ could have been toxic, leading to apoptosis.^24^

### C. Intracellular calcium response of elongated primary cilia against shear stress

**FIG. 4.**
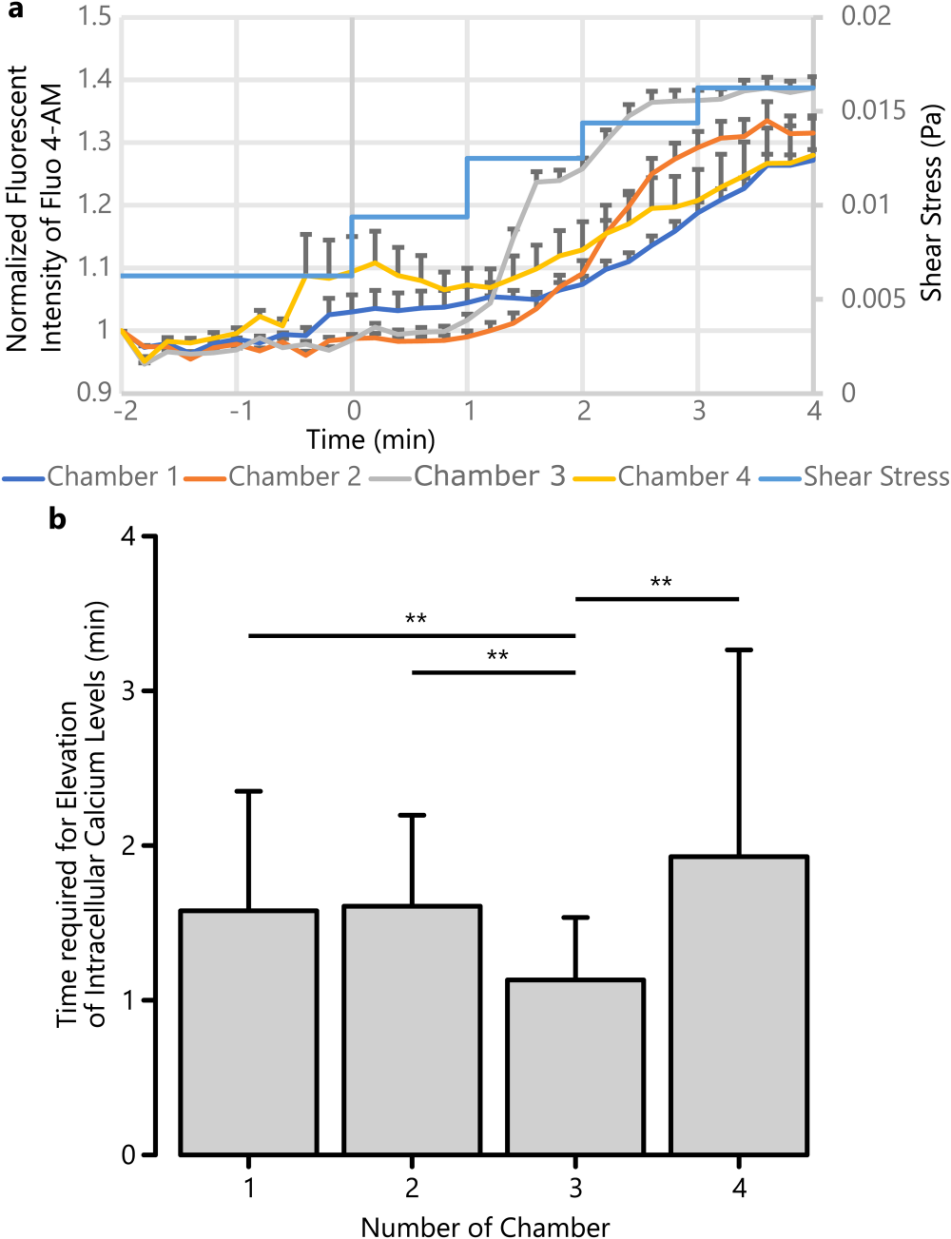
(a) The fluorescence intensity of Fluo 4-AM in each chamber during stepwise exposure of increasing shear stress. The fluorescence intensity of each chamber was standardized based on the first-time intensity. Error bars represent standard error. (b) The time required for the intracellular calcium levels to increase beyond the threshold in each chamber. **, *p* < 0.01.

To investigate the relationship between shear stress and the elongated primary cilia, we exposed primary cilia to shear stress and monitored intracellular calcium levels using fluorescent microscopy. Intracellular calcium levels of more than 25 cells in each chamber of a device were measured by the fluorescent intensity of Fluo 4-AM. Figure 4(a) shows that the intracellular calcium levels of RCTEC-DBA cells in all the chambers were increased at some time points, responding to stepwise shear stress, as RCTEC-DBA cells exposed to H_2_O_2_ responded earlier, which was measured under relatively weaker shear stress. As shown in Fig. 4(b), RCTEC-DBA cells in chamber 3 showed a significantly faster response compared to RCTEC-DBA cells in other chambers. We exposed shear stress of approximately one-tenth of the scale of shear stress present *in vivo* to mimic the acute reperfusion phase during transplantation.^25^

Based on these results, we speculate that because the shear stress was weak after ischemia-reperfusion injury, primary cilia were elongated and were more exposed to shear stress maintaining normal calcium response and homeostasis. The time lag of the increase in the intracellular calcium level between the data of chamber 3 and the data of other chambers was approximately 30 s; therefore, all of the responsive shear stress seems to be 0.0125 Pa, both in chamber 3 and other chambers. It is reported that there is a delay of approximately 20 s between shear stress stimulation and calcium response.^26^ Therefore, the time lag between the response of chamber 3 and that of other chambers indicates that the strength of shear stress was different in chamber 3 compared to that in other chambers.

## IV. CONCLUSIONS

In this study, we investigated the effect of cilia elongation from oxidative stress and the intracellular calcium levels under shear stress using a new microfluidic device. By modulating shear stress, we found that when primary cilia were exposed to oxidative stress, they showed higher sensitivity. Therefore, our microfluidic device could help in elucidating the influence of the morphology of primary cilia in low perfusion situations like acute kidney injury.

## SUPPLEMENTARY MATERIAL

See the supplementary material for the relationship between the average velocity of microspheres in a chamber and the flow rate of a syringe pump control (Fig. S1).

## Data Availability

The data that support the findings of this study are available within the article and its supplementary material.
